# Pre-diagnosis physical activity habits are associated with age of diagnosis in Parkinson's disease^☆^^☆☆^^☆☆☆^

**DOI:** 10.1016/j.prdoa.2019.07.004

**Published:** 2019-07-29

**Authors:** Merrill R. Landers, Kyle N. Johnson, Samantha Johnson, Tyler Ormsby, Danielle C. Salgo, Jessica B. Zorn, James Lyle, Andrew S. Murtishaw, Arnold M. Salazar, Jefferson W. Kinney

**Affiliations:** a Department of Physical Therapy, University of Nevada, Las Vegas, 4505 Maryland Parkway, Box 453029, Las Vegas, NV 89154, USA; b Encompass Health Rehabilitation Hospital of Las Vegas, 1250 South Valley View Blvd, Las Vegas, NV 89102, USA; c Encompass Health Rehabilitation Hospital of Henderson, Henderson, NV 89052, USA; d Custom Physical Therapy, 1450 E Prater Way Unit 103, Sparks, NV 89434, USA; e Benchmark Human Services, 11350 Random Hills Road, Suite 885, Fairfax, VA 22030, USA; f 11257 Mile 2 E., Mercedes, TX 78570, USA; g Alzheimer's Association, Chicago, IL, USA; h Department of Brain Health, University of Nevada, Las Vegas, USA

**Keywords:** Brain-derived neurotrophic factor, Exercise, Neuroprotection, Balance, Gait

## Abstract

**Introduction:**

Studies suggest that exercise may be neuroprotective when implemented before the clinical presentation of Parkinson's disease (PD). Levels of brain-derived neurotrophic factor (BDNF), theorized to play a role in neuroprotection, are affected by its genotype and exercise. Here we explore this previously unstudied interaction on age at diagnosis and severity of symptoms.

**Methods:**

76 participants with PD submitted buccal cells to determine BDNF genotype, completed the modified Lifetime Physical Activity Questionnaire to determine exercise habits, and were assessed using the Movement Disorder Society – Unified Parkinson's Disease Rating Scale III (MDS-UPDRS-III) and the Mini-Balance Evaluations Test (MBT). For aim 1 (age at diagnosis), 60 participants (age = 69.6 ± 7.4; males = 45, females = 15) were analyzed. For aim 2 (severity of symptoms), 54 participants (age = 70.0 ± 7.6; males = 41, females = 13) were analyzed.

**Results:**

The final hierarchical regression model for age at diagnosis produced an R^2^ = 0.146, *p* = .033; however, the only significant variable in the final model was average moderate physical activity from ages 20s to 40s (*p* = .009). The regression for MDS-UPDRS III was not significant; however, the regression for MBT was, *p* = .0499. In the final model, 23.1% of the variance was explained. Years since diagnosis (*p* = .014) and average vigorous physical activity from ages 20s to 40s (*p* = .047) were the only predictors in the final model.

**Conclusions:**

While a strong interaction between BDNF genotype and lifetime physical activity was not observed, our results suggest that lifetime exercise may be neuroprotective in PD. Specifically, higher amounts of moderate PA were associated with an older age at diagnosis.

## Highlights

• This is the first study to show that pre-diagnosis exercise is associated with age at diagnosis.
• Higher amounts of moderate physical activity were associated with an older age at diagnosis.
• Every hour increase in weekly moderate physical activity from ages 20–40 was associated with a PD diagnosis 3 months later.

## 1 Introduction

Several meta-analyses and systematic reviews have concluded that exercise is effective at improving many Parkinson's disease (PD) symptoms [[1], [2], [3], [4], [5]]. In addition, there are multiple compelling lines of evidence that exercise may not only provide short-term benefit to PD symptoms but it may also attenuate the degeneration [6,7]. Taken together, these lines of evidence suggest that post-diagnosis exercise is an important treatment in PD and may also help to slow PD progression.

Exercise has also been theorized to trigger several neuroplasticity events in human PD that have the potential to be neuroprotective [8,9]. Pre-clinical animal exercise studies have demonstrated mitigation of PD symptoms with concomitant changes in underlying disease processes [[10], [11], [12], [13], [14], [15], [16], [17], [18], [19]]. In human epidemiological studies, exercise performed throughout one's lifetime may decrease the risk of developing PD [[20], [21], [22], [23], [24], [25], [26]]. Collectively, these studies suggest that exercise confers neuroprotective benefit when implemented before and after the clinical presentation of PD.

One purported mechanism in PD neuroprotection is exercise-induced increases in neurotrophins, particularly brain-derived neurotrophic factor (BDNF) [[6], [7], [8], [9]]. BDNF plays a critical role in neuroplasticity [27] and exerts a protective effect on dopaminergic neurons [[28], [29], [30], [31], [32]]. BDNF levels are also lower in people with PD compared to healthy controls [33]. In addition, BDNF levels correlate with PD symptoms and motor impairment [33,34]. Not only does BDNF increase after acute exercise in healthy adults, [35] it also increases after long-term exercise programs in individuals with PD, [34,36] which suggests an upregulation BDNF gene expression. Therefore, long-term exercise represents a mechanism that influences a potentially neuroprotective biological target in PD.

A single nucleotide polymorphism in the BDNF gene (val66met, Rs6265) has functional consequences for the amount of BDNF produced. That is, the presence of a methionine allele in place of valine decreases the amount of BDNF that is released in animal models [[37], [38], [39]] and human models in response to exercise [[40], [41], [42]]. This latter study suggests that there may be an interaction between BDNF genotype and exercise such that those with a val/val genotype produce more potentially protective BDNF in response to exercise compared to met allele carriers. In addition, participants who were post-stroke with a val/val genotype had a better response to aerobic exercise and activities to promote motor learning than those with a met allele [43]. While it appears unlikely that the Val66Met polymorphism alone affects the age of onset or severity of PD progression, [30] it is possible that an interaction with history of exercise or physical activity would. However, no studies have investigated the effect of and interaction between genotype and exercise in PD on age at clinical diagnosis and disease severity.

Therefore, Aim 1 of this study was to determine if BDNF genotype interacts with lifetime self-reported physical activity (PA) levels to affect age at PD diagnosis. Aim 2 was to determine if BDNF genotype interacts with self-reported PA on disease severity as determined by measures of motor function, gait, and postural stability. We predicted that individuals with a more favorable BDNF genotype (i.e., val/val) and higher lifetime PA levels would be older at clinical diagnosis and would demonstrate fewer current PD motor symptoms, including balance and gait, after controlling for years since diagnosis.

## 2 Methods

### 2.1 Study design

This cross-sectional study involved a one-time assessment of participants with PD on the following: demographic information (age, age at diagnosis, Montreal Cognitive Assessment (MoCA), fall history, Hoehn and Yahr Stage, PD medications), self-reported lifetime PA habits, BDNF genotype, PD motor symptoms, and measures of gait and balance function. Assessments took place in an academic research laboratory, community senior centers, and participant homes from October 2016 to March 2018. The study was approved by the University of Nevada, Las Vegas Institutional Review Board.

### 2.2 Participants

**Fig. 1:**
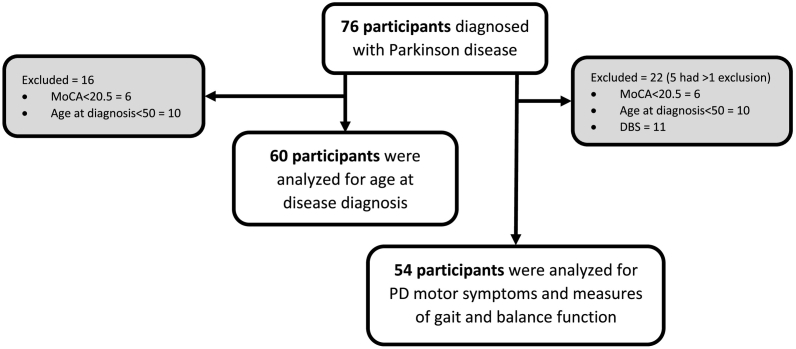
Flow diagram for participants with PD for prediction of age at PD diagnosis, PD motor symptoms, and measures of gait and balance function.

Seventy-six individuals with PD participated as a sample of convenience. To be included, participants had to be diagnosed by a neurologist with idiopathic PD. Participants with a MoCA score below 20.5 were excluded from the analysis because most of the variables were self-report and this cutoff was considered optimal for dementia in PD [44]. Additionally, the MoCA has been shown to have excellent test-retest reliability with an ICC = 0.97 and good validity for the detection of mild cognitive impairment in people with PD [45,46]. Recruitment included snowball strategies and recruiting visits to PD support groups, community gyms, senior centers, and movement disorder neurologist referrals. Because of the design of this study, two different sample sizes were used for the analyses. For Aim 1 of age at diagnosis, participants who were diagnosed with early “onset” PD (<50 years at diagnosis) were eliminated from the analysis because early diagnosis before the age of 50 would overlap with PA time frames. This resulted in 60 participants for that analysis (Fig. 1). For Aim 2 of PD motor symptoms and gait and balance, 54 participants were analyzed (Fig. 1). Because deep brain stimulation (DBS) would be potentially confounding for PD motor symptoms, participants who had had the procedure were excluded from those analyses. Please note that these exclusions were not mutually exclusive; therefore, in some cases, excluded participants may have been excluded for more than one criterion (Table 1).

**Table 1:** Demographics data for Aim 1 (age at disease diagnosis) and Aim 2 (PD motor symptoms and measures of gait and balance function).

	Aim 1 (*n* = 60)	Aim 2 (*n* = 54)
Age (years)	69.6 ± 7.4	70.0 ± 7.6
Sex	Males = 45 Females = 15	Males = 41 Females = 13
Age at diagnosis (years)	63.2 ± 8.1	63.9 ± 7.9
MDS-UPDRS III	32.7 ± 15.2	33.4 ± 15.3
MoCA	26.0 ± 2.2	26.1 ± 2.3
Levodopa equivalent dose	733.7 ± 1321.3	720.2 ± 1383.8
Hoehn and Yahr stage	1 = 4 1.5 = 1 2 = 26 2.5 = 2 3 = 22 4 = 5	1 = 2 1.5 = 1 2 = 24 2.5 = 2 3 = 21 4 = 4
Deep brain stimulation	Yes = 6 No = 54	Yes = 0 No = 54
BDNF genotype	val/val = 41 val/met = 18 refused = 1	val/val = 36 val/met = 17 refused = 1
20–40 moderate PA (hours per week)	12.0 ± 11.0	12.1 ± 10.9
20–40 vigorous PA (hours per week)	8.0 ± 9.1	7.6 ± 8.4
Moderate PA since diagnosis (hours per week)		5.6 ± 5.1
Vigorous PA since diagnosis (hours per week)		4.1 ± 5.9

### 2.3 Physical activity

PA levels were determined using a modified version of the Lifetime Physical Activity Questionnaire (LPAQ) [47]. The questionnaire includes questions about time spent sitting, walking, performing moderate activity, and performing vigorous activity during a total of seven life stages (teens, 20s, 30s, 40s, 50s, during the 5 years before PD diagnosis, and during the time since diagnosis with PD). There has been good evidence for the reliability and validity of the LPAQ as well as other similar questionnaires [48,49]. Additionally, we tested test-retest reliability from a subset of participants (*n* = 15) in this study and showed good reliability with ICCs (3,1) ranging from 0.77 to 0.91 at all of the time points between 20s to 5 years before diagnosis for moderate and vigorous PA [50]. Walking and sitting were more variable for the same time periods and were, subsequently, not used in the analyses. Reliability for the teen years was also low for all activity levels and was likewise not used in the analyses. Thus, the LPAQ reliability was good for moderate and vigorous PA at almost all time points. Based on these reliability results and also the analyses proposed, the time frames of 20s, 30s, 40s, and time since diagnosis were considered appropriate. Because of multicollinearity issues, these measures were combined into two averages: 1. average of vigorous PA from the 20s, 30s, and 40s (20–40 vigorous PA); and, 2. average of moderate PA from the 20s, 30s, and 40s (20–40 moderate PA).

### 2.4 BDNF genotype

Buccal cells were harvested using individually wrapped, sterile swabs, and stabilized using a desiccating capsule placed into a plastic tube with the swab head (T-Swab buccal swab and Slimhelix Dri-Capsules, Isohelix, Harrietsham, UK). DNA isolation was performed using commercially available reagents, and BDNF genotype was determined via polymerase chain reaction (PCR). A 10 μl PCR reaction per sample was prepared in a thin-walled, 0.2 ml PCR tube using 2× Taq Red Master mix (Genesee Scientific, El Cajon, CA, USA), 0.2 μM each of P1, P2, P3, and P4 primers, and 20 ng genomic DNA. Thermocycling condition followed a three-step PCR program: Initial denaturation at 98 °C for 3 min; 30 cycles of 95 °C for 50 s, 64 °C for 60 s, 72 °C for 60 s; and final extension at 72 °C for 2 min. PCR products were loaded in 2% agarose gel (IBI Scientific, Dubuque, IA, USA), and amplicons were resolved at 100 V for 90 min. Gel was imaged using GE Typhoon 9410 Variable Mode Imager at XXXX Genomics Core facility and band sizes were determined using 100 bp DNA ladder (Promega, Madison, WI, USA). To ensure fidelity, genotyping was replicated through an additional separate, independent PCR. All DNA assessors were blinded to the aims and purposes of the study.

### 2.5 PD motor symptoms

PD motor symptoms were measured using the motor subscale (section 3) of the Movement Disorder Society – Unified Parkinson's Disease Rating Scale (MDS-UPDRS) [51]. The MDS-UPDRS is the most widely used scale for rating the severity of PD and the validity, inter-rater and intra-rater reliability of this instrument are well established [[52], [53], [54]].

### 2.6 Gait and balance function

Gait and balance function were quantified using both a self-reported measure, the Activities-Specific Balance Confidence Scale (ABC), [55] as well as a clinician-administered measure, the Mini-BESTest (MBT) [56]. Both the ABC and the MBT have good evidence for reliability and construct validity in the PD population [[55], [56], [57], [58], [59], [60], [61]].

### 2.7 Sample size calculation

Sample size was estimated using the multiple regression module from PASS 16.0 (NCSS, LLC. Kaysville, Utah, USA, ncss.com/software/pass). For the prediction of age at diagnosis, 37 participants were needed (α = 0.05, 80% power) with one variable (BDNF genotype) conservatively estimated to have a low R^2^ of 0.150 entered first and two other variables (20–40 vigorous PA; 20–40 moderate PA) entered next at low to moderate R^2^ of 0.200. For the prediction of current PD motor symptom severity (MDS-UPDRS III), 45 participants were needed (α = 0.05, 80% power) with two variables entered first (BDNF genotype, years since diagnosis) and conservatively estimated (R^2^ of 0.150) and four other variables (20–40 vigorous PA; 20–40 moderate PA; moderate PA since diagnosis; vigorous PA since diagnosis) at low to moderate R^2^ of 0.200.

### 2.8 Data analysis

Data were analyzed using SPSS version 24.0 (SPSS Inc., Chicago, Illinois) at α = 0.05. Hierarchical linear regression analyses were conducted to examine the relationship between predictor variables (BDNF genotype and PA levels) on the following dependent variables: age at diagnosis, PD motor symptoms (MDS-UPDRS III), and measures related to gait and balance (MBT, ABC). For age at diagnosis, the following variables were entered into the hierarchical model: step 1 (BDNF genotype) and step 2 (20–40 vigorous PA; 20–40 moderate PA). For PD motor symptoms (MDS-UPDRS III) and gait and balance (MBT, ABC), the following were entered into the model: step 1 (BDNF genotype, years since diagnosis) and step 2 (20–40 vigorous PA; 20–40 moderate PA; moderate PA since diagnosis; vigorous PA since diagnosis). Dependent variable outliers, defined as those with standardized residual values above 3.3 or below −3.3, were screened for removal from the analyses (none identified). Normality, collinearity diagnostics (Variance Inflation Factor cutoff of 10), and bivariate correlations were also conducted. There were no major deviations from normality. Due to multicollinearity, the PA for the 20s, 30s, and 40s was averaged rather than entered into the regression analyses individually.

## 3 Results

### 3.1 Age of PD diagnosis

The final hierarchical regression model for age at diagnosis produced an R^2^ = 0.146, F(3,58) = 3.125, *p* = .033 (Table 2); however, the only significant variable in the final model was 20–40 moderate PA (*p* = .009). Neither BDNF genotype (*p* = .377) or 20–40 vigorous PA (*p* = .664) were statistically significant. When 20–40 vigorous PA was removed from the model, the final model R^2^ was 0.143 and was still significant (*p* = .013). When BDNF genotype was removed from the model, the final R^2^ decreased to 0.115 but was still significant (*p* = .008).

**Table 2:** Coefficients for regression model on age at PD diagnosis.

Model		R^2^	Unstandardized coefficients		Standardized coefficients	*t*	*p* value
			B	Standard error	Beta		
1	(Constant)	0.024	66.450	3.123		21.277	0.000
	BDNF genotype		−2.669	2.257	−0.155	−1.183	0.242
2	(Constant)	0.146	62.631	3.329		18.813	0.000
	BDNF genotype		−1.928	2.166	−0.112	−0.890	0.377
	Moderate PA 20s to 40s		0.270	0.100	0.372	2.701	0.009
	Vigorous PA 20s to 40s		−0.052	0.120	−0.060	−0.437	0.664

### 3.2 PD motor symptoms

The hierarchical regression for MDS-UPDRS III was not statistically significant, F(6,52) = 0.867, *p* = .526. In the final model, none of the variables were statistically significant (ps ≥ 0.140).

### 3.3 Gait and balance function

The final hierarchical regression for MBT was statistically significant, F(6,52) = 2.304, *p* = .0499. In the final model, 23.1% of the variance was explained (R^2^ = 0.231; Table 3). Years since diagnosis (*p* = .014) and 20–40 vigorous PA (*p* = .047) were statistically significant predictors in the final model. For the ABC, the final hierarchical model explained 24.7% of the variance, F(6,52) = 2.508, *p* = .035 (Supplement A). In the final model, the only variable that was statistically significant was years since diagnosis (*p* = .002).

**Table 3:** Coefficients for regression model on MBT score.

Model		R^2^	Unstandardized coefficients		Standardized coefficients	*t*	*p* value
			B	Standard error	Beta		
1	(Constant)	0.096	23.703	2.586		9.166	0.000
	Genotype		0.836	1.658	0.068	0.504	0.616
	Years since diagnosis		−0.394	0.177	−0.300	−2.228	0.030
2	(Constant)	0.231	26.371	2.871		9.186	0.000
	BDNF genotype		0.574	1.686	0.047	0.340	0.735
	Years since diagnosis		−0.444	0.173	−0.338	−2.562	0.014
	Moderate PA 20s to 40s		−0.100	0.076	−0.190	−1.327	0.191
	Vigorous PA 20s to 40s		−0.219	0.107	−0.320	−2.037	0.047
	Moderate PA since diagnosis		0.112	0.189	0.098	0.590	0.558
	Vigorous PA since diagnosis		0.062	0.165	0.063	0.373	0.711

## 4 Discussion

Our results suggest that self-reported moderate PA during the 20s, 30s, and 40s is associated with age at diagnosis. Specifically, those who reported having spent more time in moderate PA during those three decades were older at diagnosis. This is consistent with the notion that exercise may be neuroprotective in PD and is consistent with other studies that have shown that exercise/physical activity is associated with a lower risk of PD [[20], [21], [22], [23], [24], [25], [26]]. Our results suggest that every hour increase in weekly moderate PA (unstandardized beta = 0.270) was associated with a PD diagnosis a little over a quarter of a year later. While this relationship is potentially important from a neuroprotective perspective, it should be noted that this only explained 14.6% of the variance of age which suggests that a larger portion of the variance remains unexplained by our model. We had anticipated that BDNF might play a role in potentiating the neuroprotection but the low and non-significant correlations, though headed in the correct direction (i.e., val/val carriers having an older age at diagnosis), suggests that BDNF genotype may only play a minor role.

Other studies have demonstrated similar neuroprotective effects of exercise in PD [20,21,23,62]. For example, Chen et al. reported that men who performed greater baseline PA had a decrease risk of PD [20]. However, contrary to our findings, further analysis by Chen and colleagues revealed that only vigorous and not moderate activity was related to the lower risk of PD in their male participants [20]. It is important to note that their participants were aged 30–55, which is an older and different lifespan period than our study. Sääksjärvi et al. reported that, compared to those who engaged in no heavy leisure-time activity, individuals who performed heavy PA were less likely to develop PD [23]. In their study, heavy leisure-time activity was defined as doing >3 h per week of activities such as jogging, skiing, or vigorous gardening [23]. A slight protective effect of PA was also described by Sasco et al., who found that belonging to a varsity team in college was associated with a lower nonsignificant risk of PD [21]. Moderate PA done during adulthood was also found to be linked to a reduced risk of PD [21]. Yang et al. reported an inverse association between PA and PD as well. However, their team looked at total PA as compared to the other studies that focused on moderate and vigorous PA [26]. While the aforementioned studies reported a similar construct, our study differs in that those studies were large retrospective cohort studies wherein a cohort of healthy individuals were tracked over time to determine who would eventually develop PD. Thus, they looked at how PA influenced the risk of PD. We used a cross-sectional design wherein only individuals already diagnosed with PD were included, examining the potential benefits of pre-diagnosis PA for those who ultimately do develop PD. While each of these epidemiologic studies in isolation, including the present study, is not strong for causal inference of neuroprotection in PD, the evidence for causal inference is enhanced by the number of studies supporting the construct, the consistency of results across different designs, the strength of associations, and the biological plausibility.

While moderate PA was predictive of age at diagnosis, vigorous activity was not. On the surface, it seems logical that vigorous activity, much like moderate PA, would be associated with age at diagnosis. However, it is possible that vigorous activity, which is typically close to or over the anaerobic threshold, may produce more pro-inflammatory signals thereby increasing the inflammatory milieu. Since inflammation is a known pathophysiologic mechanism [63,64] and a potential disease trigger, [65] it is possible that engaging in high amounts of vigorous exercise may tip the inflammatory balance to a potentially deleterious process. Thus, the issue of dosing warrants additional scientific exploration particularly as it relates to inflammatory biomarkers (e.g., cytokines, acute phase proteins).

None of the predictor variables in our study were associated with current PD motor symptoms as measured by MDS-UPDRS-III score. We had anticipated that greater PA and a favorable BDNF genotype (i.e., more BDNF produced with val/val genotype relative to genotypes with a met allele [[40], [41], [42]]) would have produced a better neuroprotective environment [64,66,67] which presumably would have strengthened neural networks associated with motor function. This, in turn, was hypothesized to promote a slower and more protracted decline in motor function. However, our results did not support this notion.

Another noteworthy finding of this study is that two variables were significantly associated with gait and balance. First, we found that years since diagnosis was negatively correlated with balance. Essentially, the longer an individual had been diagnosed with PD, the worse their gait and balance. This is logical and consistent with the literature [68,69]. Second, there was a negative correlation between MBT score (higher score equates to better balance function) and time spent in vigorous PA during their 20s–40s which means that poorer balance performance was associated with higher amounts of vigorous PA during their 20s–40s. This finding is counter intuitive and does not fit our original hypothesis, which was that more PA (moderate and/or vigorous) would positively affect balance later in life. The strength of the association suggests that 1 additional hour of vigorous PA per week would be associated with a 0.22 decrease in MBT score which is considerably lower than the 5.52 minimal detectable change in PD [59]. Thus, even if there was indeed a cause and effect relationship, the effect is quite small. On the other hand, it is also possible that this relationship is spurious in that there may be a latent factor mediating the relationship. Future research will need to have more sophisticated designs to further explore this relationship.

Self-reported levels of PA were remarkably high for the sample with 78% to 89% of the participants reporting having met Centers for Disease Control recommendations for PA [70] at all stages of their lives. In comparison, only 51% of adults in the United States report meeting CDC guidelines [71]. Additionally, older adults tend to over-estimate physical activity [72,73]. A study by Tucker et al. found that while 62% reported meeting 2008 PA Guidelines, only 9.6% truly met the guidelines as measured by accelerometer, a pattern that was consistent at all age groups (20s, 30s, 40s, 50s, 60s, >70) [74]. Thus, it is possible that participants in the present study also overestimated their PA levels and/or the sample may have been biased by volunteers who were more likely to be physically active. On the other hand, it is possible that participants at both ends of the physical activity spectrum exaggerated their activity levels relatively equally (non-differential misclassification bias). In addition, it is possible that participants exaggerated their PA levels to avoid looking inactive to the research assistants, thinking that by reporting their actual levels they might be perceived as bearing partial responsibility for their PD. This type of participant bias is referred to as social desirability bias and it has been documented in the self-report of physical activity [75]. Lastly, it is important to bear in mind that PA also reflects work and it is possible that participants may have had occupations that required considerable PA.

An important limitation was that we could not verify if the PD diagnoses were made using well-established clinical criteria and we could not verify that the diagnoses were made by qualified neurologists. We depended solely on the veracity of the participant self-report of neurologist diagnosis. Another limitation is that we used age of diagnosis instead of age of onset. The reason is that we felt it would be easier to remember the actual year of diagnosis and would be less susceptible to recall bias than their “best guess” of disease onset. Another study limitation is that the buccal collection method may not have provided the ideal yield of usable DNA. Moreover, oral health and medication may negatively affect the quality of the DNA. However, the quality and quantity of the DNA extracted through buccal collection was sufficient to run each sample in duplicate in all cases.

## 5 Conclusion

Our results suggest that people with PD who reported more moderate PA from the 3rd through 5th decades of life were older at diagnosis than those who reported lower levels of PA. BDNF did not appear to play a large role in age at diagnosis or current PD motor symptoms.

## Declaration of competing interest

The authors does not have any conflict of interest.
